# Effect of wheat bran dietary fiber on structural properties and hydrolysis behavior of gluten after synergistic fermentation of *Lactobacillus plantarum* and *Saccharomyces cerevisiae*

**DOI:** 10.3389/fnut.2022.982878

**Published:** 2022-09-20

**Authors:** Zhen Wang, Sen Ma, Li Li, Jihong Huang

**Affiliations:** ^1^State Key Laboratory of Crop Stress Adaptation and Improvement, College of Agriculture, Henan University, Kaifeng, China; ^2^College of Food Science and Engineering, Henan University of Technology, Zhengzhou, China; ^3^School of Food and Pharmacy, Xuchang University, Xuchang, China

**Keywords:** dietary fiber, sourdough, gluten, protein hydrolysis, FT-IR

## Abstract

The effect of synergistic fermentation of *Lactobacillus plantarum* and *Saccharomyces cerevisiae* on the structural properties and aggregation behavior of gluten containing different wheat bran dietary fiber (WBDF) levels (0, 3, 6, 9, and 12%) was investigated. The results showed that WBDF addition affected the aggregation behavior of gluten at the molecular level, while WBDF significantly induced depolymerization behaviors in large aggregated gluten proteins (Molecular weight > 130 kDa) under reducing conditions (*p* < 0.05). In terms of secondary structure, WBDF significantly reduced glutamine side chain levels and reduced antiparallel β-sheet structures from 28.57 to 24.53% (*p* < 0.05). In addition, WBDF thermal properties and its water holding capacity were the main factors causing changes in thermal properties in the overall gluten system. This study provides new data for the improved production of sourdough whole grain and/or high fiber flour products.

## Introduction

The consumption of more whole grain foods, such as wholemeal bread, may help reduce diseases such as type 2 diabetes, chronic cardiovascular disease, chronic intestinal disease, and obesity (1, 2) as whole grain foods are rich in active substances that benefit human health, especially dietary fiber (3). However, wholemeal flour products have a poorer texture and mouth-feel when compared with refined flour products; one reason is the increased insoluble dietary fiber content in wholemeal products which deteriorates gluten network structures during flour production processes, ultimately leading to reduced quality in flour products (4).

Sourdough fermentation technology is considered a promising way to improve wholemeal product quality; it is considered a “gold standard” fermentation technique and an effective way to meet future food challenges (5, 6). However, the use of sourdough in industrial fermentation processes is challenging as microorganism complexity in sourdough is significantly influenced by the environment (7). This potentially causes difficulties with product quality control.

In general, sourdoughs are classified as Types I, II, and III depending on how the culture was obtained. Type I sourdoughs are traditional in nature and require uninterrupted propagation through the regular application of fresh flour and water. Type II sourdoughs are industrially inoculated with adapted cultures which function as dough acidifiers and Type III sourdoughs are usually dried for easy storage and use (8, 9). Therefore, type II are considered the best options for factory production processes as consistent controlled quality can be ensured. And the most important step before producing type II sourdough is to select the right strains to build a synergistic fermentation system.

On the other hand, one of the most typical features of sourdough fermentation is gluten hydrolysis (10), the degree of which is thought to correlate with gluten structure, flavor precursors, dough quality, and flour product quality. For example, the flavor of wheat sourdough bread is positively correlated with the concentration of free amino acids during production (11), and increased free amino acids are generated by gluten hydrolysis during fermentation. Monitoring gluten behavior during sourdough fermentation is a main research focus; however, sourdough composition is also very important. Lactic acid bacteria (LAB) and yeast (mainly *Saccharomyces cerevisiae*) are the most important microorganisms in sourdough production (12); one of the best-known LABs is *Lactobacillus plantarum* which is a homo-fermented LAB (13). In the LAB system, lactic and acetic acids are, respectively, produced *via* specific metabolic hexose and pentose pathways; the acids lower the pH of the fermentation system and inhibit ammonia nitrogen production. Conversely, *S. cerevisiae* secretes nitrogen-metabolizing enzymes to hydrolyze exogenous amino acids and peptides to inorganic nitrogen (9, 14).

Currently, data on the effects of cereal dietary fiber in sourdough fermented flour products are often contradictory, especially with regard to underlying mechanisms. In this study, dietary fiber was extracted from wheat bran and a synergistic fermentation system constructed using *L. plantarum* and *S. cerevisiae* after separate immobilization cultures. Our aim was to investigate the influence of wheat bran dietary fiber (WBDF) on structural properties and aggregation behavior of gluten in the synergistic fermentation system, with a view to providing additional data for research and the industrial production of whole grain and/or high dietary fiber fermented flour products.

## Materials and methods

### Materials

Wheat bran was obtained from Henan Jiaqi Industry Co. (<city>Zhengzhou</city>, China), the moisture content of the bran was 14.68%, the protein content 16.51%, the starch content 12.04% and the ash content 5.89%. Raw wheat gluten was purchased from a local market (73.64% protein, 8.48% starch, 1.26% ash, and 12.07% moisture). WBDF was extracted following a procedure by Ma et al. (15); total dietary fiber, moisture, protein, starch and ash content in the WBDF was 85.90, 5.24, 2.72, 3.22, 1.75%, respectively. Finally, *L. plantarum* ATCC 8014 and *S. cerevisiae* ATCC 9763 were provided by the Shanghai Biology Collection Center (Shanghai, China). All reagents were analytically pure unless otherwise specified.

### Preparation of WBDF-gluten samples

WBDF was mixed with raw gluten (RG) at 0, 3, 6, 9, and 12% (*w/w*) concentrations in a mixer (JF-300 Rotary Mixer, Worcestershire Industrial Instruments Ltd., Guangzhou, China) at 100 rpm for 5 min to generate WBDF-gluten samples (Control-0, 3, 6, 9, and 12%).

### Preparation of a synergistic fermentation system

*L. plantarum* ATCC 8014 and *S. cerevisiae* ATCC 9763 were cultured separately to construct a type II sourdough fermentation system, with the ultimate goal of generating a mixed microorganism suspension. *L. plantarum* and *S. cerevisiae* levels were log 9.0 CFU/mL and log 7.0 CFU/mL, respectively. All steps were performed according to a previous study (16). LAB and yeast cell counts were performed according to the AOAC 990.12 method.

### The fermentation of WBDF-gluten samples

WBDF-gluten samples and the sourdough were mixed in a 1:2 (*w/v*) ratio. Next, the gluten slurry were transferred to a constant temperature and humidity incubator (Shanghai Binglin Electronic Technology Co., Ltd., Shanghai, China) with a pre-set temperature of 30°C, humidity of 80%; and the fermentation time lasted 4 h. Finally, the fermented gluten slurry was centrifuged for 30 min at 4,000 × *g*. The precipitates were collected, freeze-dried for 48 h, and ground to enable all samples to pass through a 100 mesh sieve; stored at −18°C.

### Scanning electron microscope analysis

The structure of WBDF-gluten fermentation samples was examined by SEM. All samples were sputtered for gold plating in a sputtering coater and transferred to a Quanta FEG-250 SEM (FEI, Hillsboro, OR, USA) for examination at an accelerated voltage of 20 kV.

### Confocal laser scanning microscopy analysis

All samples need to be dyed before CLSM observation. The fermented WBDF-gluten samples were mixed and dyed with fluorescent dye rhodamine B [0.001% (*w/v*)], fluorescein isothiocyanate (FITC) [0.01% (*w/v*)] and fluorescent brightener [0.001% (*w/v*)] for 20 min. The stained sections were observed in Olympus Fluoview FV30000 CLSM system (Olympus Corp., Tokyo, Japan), and the laser excitation wavelength was set to 561 nm. Digital image files were recorded at a resolution of 1,024 × 1,024 pixels.

### Determining the molecular weight distribution

WBDF-gluten and RG samples were weighed (150 mg) and dissolved in 15 mL 0.05 M phosphate buffer [pH 6.8, 2.0% (*w/v*) plus sodium dodecyl sulfate (SDS)] and vortexed at 25°C for 30 min. Sample suspensions were then centrifuged at 25°C for 15 min at 10,000 × *g* and supernatants passed through a 0.45 μm aqueous filter membrane. A BioSP-sec-s4000 liquid chromatography (LC) column was selected for size exclusion high performance liquid chromatography (SE-HPLC). Experimental LC conditions were: column temperature = 30°C; mobile phase A = acetonitrile (chromatographic grade) + 0.05% trifluoroacetic acid, mobile phase B = H_2_O (ultrapure water) + 0.05% trifluoroacetic acid; mode = equal gradient (A:B = 1:1); injection volume = 20 μL; flow rate = 0.5 mL/min; and detection wavelength = 214 nm. In reducing conditions, the procedure was the same except dithiothreitol (DTT) was added.

### Fourier transform infrared spectroscopy analysis

FT-IR spectra were generated using a fast FT-IR spectrometer. Samples (2 mg) were ground for 5 min with 200 mg dry potassium bromide. Test conditions: scanning range = 4,000 cm^−1^-400 cm^−1^, resolution = 4 cm^−1^, and number of scans = 32. Spectral data were processed using Peakfit software (v4.12) in the amide I band (1,650–1,720 cm^−1^) range to analyze secondary protein structures. Specific structures attributed to different spectra in the amide I band are shown (Supplementary Table S1).

### Amino acid analysis

The free amino acid content in samples was analyzed using a fully automatic amino acid analyzer (Clarity Amino Acid Analyzer SW-433D, Sykam, Munich, Germany); 150 mg samples were weighed, extracted in boiling water, and separated on a sulphonic acid cation exchange column. Then, at 135°C, separated amino acids were reacted with ninhydrin: primary amines produced blue-violet compounds and secondary amines produced yellow compounds. Derivatives were detected by visible spectrophotometric analyses at 570 and 440 nm, respectively, using a chemical reaction. Amino acids were characterized by sample retention times and quantified using an external standard working curve.

### Surface hydrophobicity tests

Protein surface hydrophobicity (H_o_) was determined using the fluorescent probe 8-anilino-1-naphthalenesulfonic acid (ANS). WBDF-gluten samples were weighed (100 mg) and dispersed in 15 mL 0.01 M phosphate buffer (pH 7.0). The dispersion was shaken and centrifuged at 10,000 × *g* for 10 min at 4°C. Next, the protein concentration of the supernatant was determined using the Bradford method, with or without dilution as appropriate. Approximately 4 mL of the supernatant was set aside for assay. An ANS solution (8 mM) was prepared in the same phosphate buffer and 20 μL added to the 4 mL supernatant and fluorescence intensity measured after incubation for 3 min. The excitation wavelength was 390 nm, the emission wavelength was 470 nm, and excitation and emission slits were 5 nm. is the H_o_ value of the sample, and a diluted gluten solution without ANS was used as a blank control.

### Thermal properties

All samples were placed in aluminum oxide trays after accurate weighing of 5 mg. Thermogravimetric analysis (TGA) of the samples was carried out with a Mettler Toledo TGA2 (SF) system (Mettler Toledo Corp., Zurich, Switzerland). The experimental conditions were set to a heating rate of 10°C/min from 30 to 600°C and a nitrogen atmosphere flow rate of 60 mL/min. Derivative thermogravimetric (DTG) curves were obtained by performing first-order derivative analysis on TGA trace mass loss (%).

### Statistical analysis

All procedures were repeated three times. Analysis of variance was used to detect significant differences (*p* < 0.05). One-way analysis of variance was performed using Tukey's method in SPSS software (SPSS Inc., Chicago, IL, USA).

## Results and discussion

### Morphology analysis

**Figure 1:**
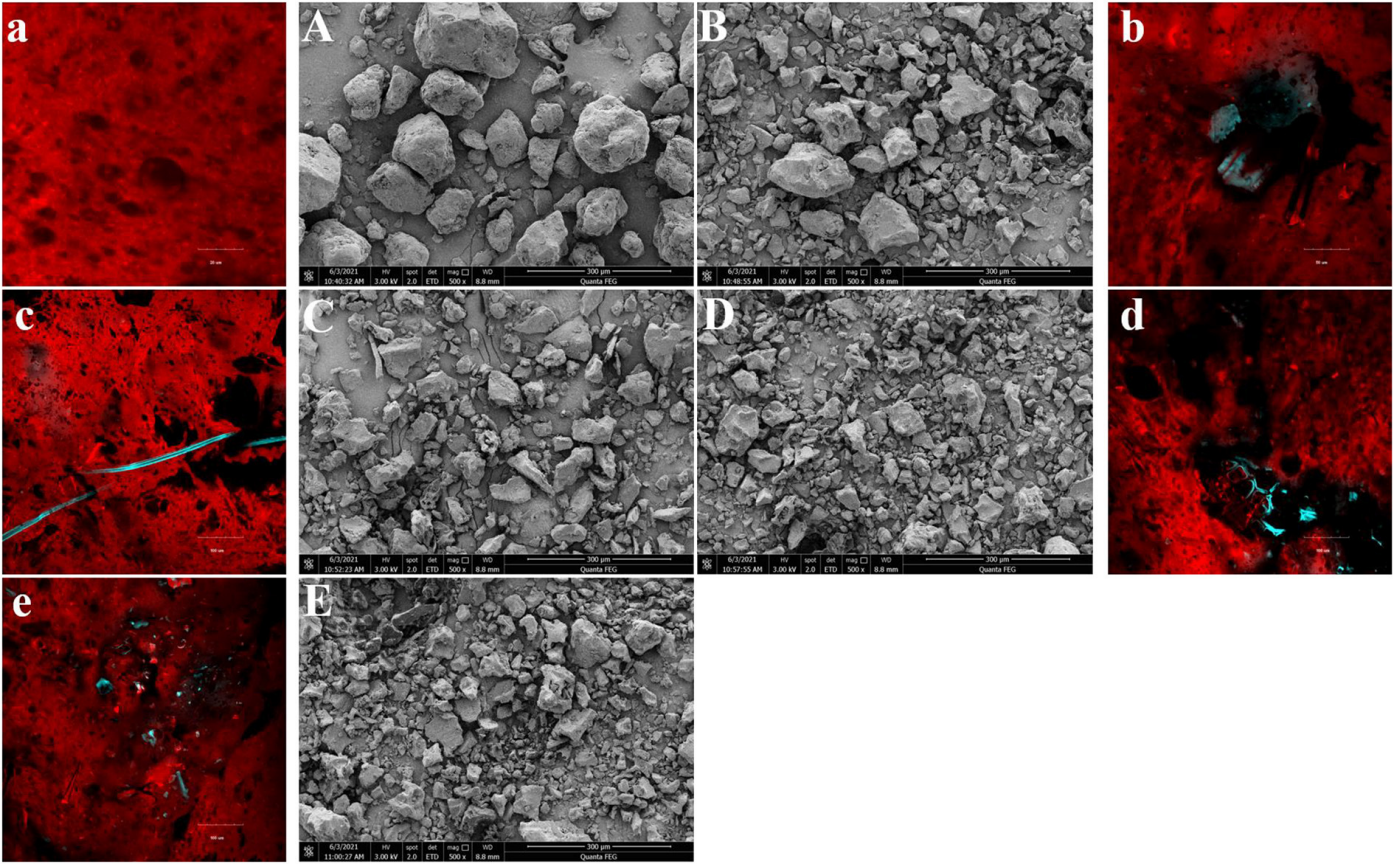
The surface morphology of samples at different WBDF levels (0%-Control, 3%, 6%, 9%, 12%) was observed by SEM (500 ×, **A–E**) and CLSM **(a–e)**.

The surface morphology of gluten after fermentation at different WBDF concentrations is shown (Figure 1). SEM images (Figures 1A–E) showed that gluten was more visible as agglomerates (clumps), especially in control samples (Figure 1A). In WBDF-gluten samples (Figures 1B–E), most of the WBDF was encapsulated by gluten. Also, the particle size of gluten agglomerates decreased with increasing WBDF concentration and was accompanied by rougher protein agglomerate surfaces and deepened protein fragmentation, especially in 9 and 12% WBDF samples (Figures 1D,E).

After fermentation, circular or elliptical air cells inside gluten were observed in CLSM images (Figures 1a–e) and WBDF positions in relation to air chamber formation were clearly seen. In this case, the gluten is stained red by rhodamine B; the WBDF is stained blue by FITC; the fluorescent brightener serves to make the imaging results of the WBDF clearer. In control samples (Figure 1a), gluten displayed homogeneous textures and the edges of air cells formed by fermentation were flat and smooth; whereas after WBDF addition (Figures 1b–e), the gluten matrix could barely coexist with WBDF and tore the gluten matrix. In other words, the gluten matrix was uneven and discontinuous around the WBDF; therefore, good air cell structures were not identified in WBDF samples.

Therefore, the addition of WBDF changed the distribution of gluten. Gluten is more likely to aggregate on the WBDF, encapsulating the WBDF. The degradation behavior of dough and flour products caused by insoluble dietary fiber is well-known, with several hypotheses promoted in previous reports: (1) WBDF is stiff and directly affects the rheological behavior of the dough; (2) WBDF dilutes the relative gluten and starch content and breaks the tightness of the gluten-starch matrix; (3) WBDF has a “competitive hydration” effect, which affects the structure of the gluten network and gluten hydration; and (4) WBDF forms a physical barrier to the gluten network (steric hindrance effects) and accelerates CO_2_ escape through the spatial barrier effect (4). Here, we provide new observations potentially explaining dough quality deterioration caused by insoluble dietary fiber.

### Molecular weight distribution

**Figure 2:**
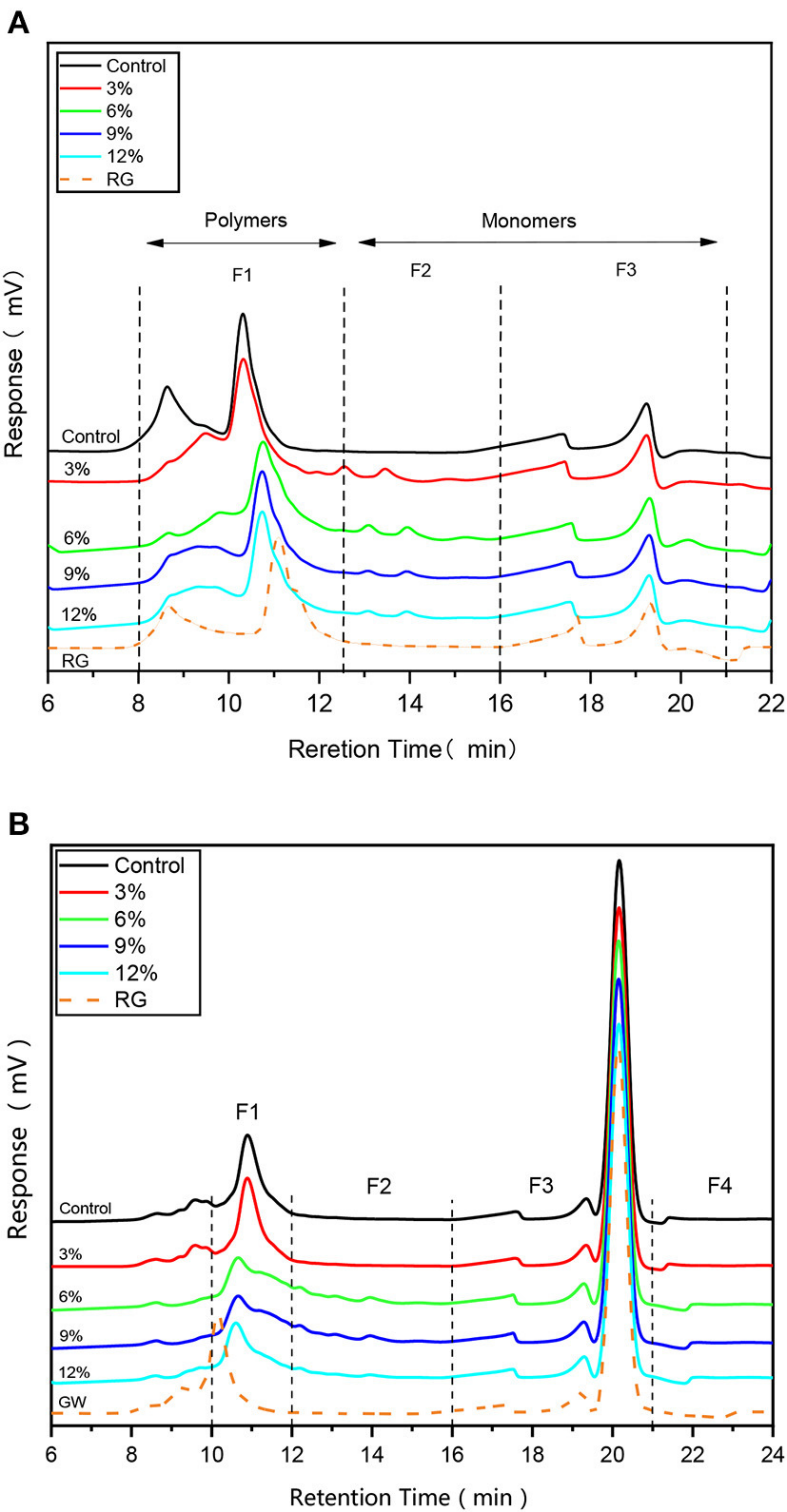
HPLC profiles of samples containing different WBDF levels under non-reducing condition **(A)** and reducing condition **(B)**. RG is raw gluten unfermented.

SE-HPLC is a sensitive method that quantifies Molecular weight (*Mw*), while gluten solubility in SDS-phosphate buffer reveals the extent of their cross-linking capabilities. Typically, in the absence of strong reducing agents (Figure 2A), SDS soluble proteins are divided into three regions based on band intensity: F1, F2, and F3, which correspond to *Mw* > 67 kDa, *Mw* ≈ 37–60 kDa, and *Mw* ≈ 14–27 kDa, respectively (17). As shown (Figure 2A), fermentation and WBDF addition altered sample retention times in the F1 region, while samples containing WBDF showed two small peaks in the F2 region, with no significant shifts in the F3 region. The higher WBDF levels in the F1 region, the closer the peak band positions were to original unfermented gluten samples, i.e. WBDF addition caused the *Mw* of WBDF-gluten aggregates to be closer to the original gluten sample, which may be related to WBDF particle size. Gluten proteins exhibit entrapment behavior for WBDF as observed in morphology. At the same time, this behavior may have an impact on the aggregation behavior of gluten and thus on the *Mw* of gluten proteins, as the contact possibilities between gluten proteins are amplified. The particle size of WBDF may form the basis for the influence of gluten *Mw*. No significant decrease in band intensity was observed in any plots; however, in the F1 region, the peak near the 8.5 min retention time had shifted in the WBDF sample when compared with the control sample and the original gluten sample, and was supported by a small peak in the F2 region. This observation suggested that WBDF addition promoted a slight hydrolysis in protein bulk polymers in samples. The low intensity of the hydrolysis process was attributed to the short fermentation time; in an optimal fermentation period (3–5 h) LAB cannot exert sufficient effects on protein hydrolysis. In contrast, if fermentation times are extended (> 24 h), then significant protein degradation by LAB metabolites may occur, especially for malt alcohol proteins of *Mw* 30–38 kDa (17, 18).

However, upon the addition of a strong reducing agent such as DTT (Figure 2B), gluten was divided into four main intervals based on retention times of different *Mw* proteins. Specifically, proteins were divided into intervals based on 10–12 min, 12–16 min, 16–21 min, and >21 min retention times and corresponded to F1, F2, F3, and F4 intervals (Figure 2B), respectively (19). F1 corresponded to larger polymeric proteins (*Mw* > 130 kDa), F2 to smaller polymeric proteins (*Mw* = 80–130 kDa), F3 to larger monomeric proteins (*Mw* = 10–80 kDa; mainly gliadins), and F4 to smaller monomeric proteins, peptides, and amino acids (*Mw* < 5 kDa). As shown (Figure 2B), the most significant protein band changes were identified in the F1 region, particularly in 6, 9, and 12% WBDF samples, which showed a significant decrease in the F1 peak (*p* < 0.05) and indicating a decrease in protein aggregates in this region. Apart from this observation, bands in other profile regions were not changed significantly and remained consistent with correlation trends between the *Mw* and WBDF levels in samples. This result shows that the addition of WBDF does not alter the aggregation state of gluten proteins at the level of disulfide bonds. At the same time, previous studies have shown that the solubility of gluten proteins is strongly altered by sourdough fermentation, but has not been able to demonstrate that proteolysis of gluten proteins to water-soluble amino acids or peptides occurs (10).

### Secondary structures

FT-IR methods are widely used to study protein secondary structures. The basic principle is to divide the spectrogram into amide I, II, and III bands based on wavelength intervals, with different chemical functional groups of proteins in different bands with different stretching and vibrating modes. The amide I band (1,720–1,570 cm^−1^) is widely used as it has a significant protein signal, and amino acid side chains in this region exert less influence on results; protein signals in amide I bands mainly originate from C=O stretching vibrations in the amide group (20).

Secondary structure data from gluten samples with different WBDF concentrations after fermentation are shown (Table 1). β-sheet and α-helix structures were predominant in protein samples, consistent with previous results (21). For protein intermolecular β-sheet, α-helix, and β-turn structures, WBDF addition did not significantly affect their content (*p* > 0.05), i.e., WBDF induced no changes in these secondary structures. The most significant difference occurred in the control sample, low glutamine side chain (2.66%) levels were observed after fermentation (*p* > 0.05). Glutamine is a hydrophilic amino acid with an isoelectric point of 5.65 and an R-group composition of -(CH_2_)_2_-CONH_2_. However, the glutamine side chain structure was not observed in WBDF samples, so it was likely that the WBDF side chain, in an acidic environment created by fermentation, was non-covalently bound to the glutamine side chain. Additionally, WBDF addition significantly decreased antiparallel β-sheet levels from 28.57% in the control sample to 24.53% in the 12% WBDF sample (*p* < 0.05). Protein secondary structures are primarily maintained by non-covalent forces, and when the system environment is altered (e.g., pH, temperature, polar/non-polar co-existing species) the protein state changes accordingly. However, it should be noted, no credible reports exist on intermolecular interaction mechanisms between dietary fiber and anti-parallel β-sheets in gluten.

**Table 1:** Secondary structure contents of fermented gluten at different WBDF levels.

**Samples**	**Glutamine side chain**	**Intermolecular β-sheets**	**Antiparallel β-sheets**	**α-helices**	**β-turns**
Control	2.66 ± 0.01^a^	16.04 ± 0.01^a^	28.57 ± 2.87^a^	39.81 ± 7.83^a^	22.28 ± 4.39^a^
3%	n.d.	18.29 ± 0.00^a^	27.46 ± 0.06^a^	40.35 ± 6.35^a^	23.04 ± 2.86^a^
6%	n.d.	15.00 ± 2.00^a^	24.76 ± 0.22^b^	38.62 ± 1.74^a^	21.63 ± 0.48^a^
9%	n.d.	19.78 ± 3.57^a^	24.93 ± 0.06^b^	38.35 ± 1.83^a^	21.26 ± 0.00^a^
12%	n.d.	16.84 ± 0.32^a^	24.53 ± 0.90^b^	37.26 ± 1.06^a^	21.39 ± 0.17^a^

Different letters in the superscript indicate significant differences in the same column (*p* < 0.05). n.d., not detected.

### Free amino acid type and content

In contrast to dry yeast fermentation, the most distinctive feature of sourdough fermentation is that gluten undergoes varying levels of hydrolysis during fermentation. Protein hydrolysis, followed by peptide or amino acid metabolism *via* LAB fermentation, is a key pathway for bread flavor formation (22). Previous studies reported that fermented sourdoughs contained lower peptide and higher amino acid levels (23). Also, amino acid type and content after hydrolysis was determined by *Lactobacillus* strain specificity in sourdough (22).

As indicated (Table 2), no significant change in the relative amino acid content in samples was observed, except for serine and tyrosine. This observation could be attributed to the uniqueness of the Lactobacillus strain used in this study. Specifically, for serine and tyrosine content, no linear pattern was observed in their relationship with WBDF content, therefore, it was not possible to ascribe a mechanism to these amino acid alterations. It is generally accepted that the amount and type of amino acids produced by the hydrolysis of gluten proteins during sourdough fermentation depends on the microbial composition that makes up the sourdough. In the case of lactic acid bacteria and yeasts in sourdough, higher levels of amino acids are required for the growth and metabolism of yeasts. Previous studies reported that the effects of LAB toward total amino acid levels were insignificant, and that amino acid type and content was determined by the pH and microbial metabolism levels during fermentation; proline formation was favored at pH > 5.5 and phenylalanine, leucine, and cysteine release mainly occurred at lower pH values (11).

**Table 2:** Changes in free amino acid contents in the fermented system at different WBDF levels.

**Amino acids**	**Amino acids contents (%)**				
	**Control**	**3%**	**6%**	**9%**	**12%**
Asp	2.46 ± 0.17^a^	2.57 ± 0.07^a^	2.53 ± 0.02^a^	2.47 ± 0.06^a^	2.33 ± 0.16^a^
Thr	1.96 ± 0.08^a^	1.94 ± 0.19^a^	1.78 ± 0.09^a^	1.86 ± 0.08^a^	1.75 ± 0.06^a^
Ser	3.38 ± 0.23^ab^	3.59 ± 0.08^a^	3.35 ± 0.18^ab^	3.41 ± 0.11^ab^	3.11 ± 0.09^b^
Glu	26.02 ± 1.11^a^	27.90 ± 0.67^a^	26.10 ± 2.39^a^	26.46 ± 0.52^a^	24.72 ± 0.41^a^
Gly	2.65 ± 0.25^a^	2.85 ± 0.05^a^	2.72 ± 0.26^a^	2.71 ± 0.01^a^	2.48 ± 0.12^a^
Ala	2.05 ± 0.21^a^	2.23 ± 0.04^a^	2.11 ± 0.20^a^	2.11 ± 0.00^a^	1.95 ± 0.12^a^
Cys	0.81 ± 0.09^a^	0.89 ± 0.12^a^	0.89 ± 0.15^a^	1.01 ± 0.10^a^	0.97 ± 0.01^a^
Val	2.90 ± 0.26^a^	3.13 ± 0.05^a^	2.92 ± 0.32^a^	2.96 ± 0.05^a^	2.73 ± 0.10^a^
Met	1.11 ± 0.09^a^	1.18 ± 0.03^a^	1.09 ± 0.11^a^	1.08 ± 0.10^a^	1.03 ± 0.02^a^
Ile	2.67 ± 0.30^a^	2.87 ± 0.03^a^	2.69 ± 0.31^a^	2.76 ± 0.01^a^	2.70 ± 0.07^a^
Leu	4.84 ± 0.48^a^	5.20 ± 0.16^a^	4.88 ± 0.47^a^	5.01 ± 0.02^a^	4.66 ± 0.07^a^
Tyr	2.70 ± 0.22^ab^	2.85 ± 0.00^ab^	2.90 ± 0.13^a^	2.78 ± 0.06^ab^	2.48 ± 0.22^b^
Phe	3.63 ± 0.34^a^	3.87 ± 0.04^a^	3.70 ± 0.41^a^	3.73 ± 0.05^a^	3.41 ± 0.23^a^
His	2.07 ± 0.23^a^	2.19 ± 0.06^a^	2.12 ± 0.26^a^	2.13 ± 0.00^a^	1.98 ± 0.10^a^
Lys	1.18 ± 0.12^a^	1.25 ± 0.03^a^	1.17 ± 0.11^a^	1.18 ± 0.00^a^	1.08 ± 0.05^a^
Arg	2.58 ± 0.24^a^	2.75 ± 0.04^a^	2.60 ± 0.27^a^	2.62 ± 0.04^a^	2.42 ± 0.13^a^

Different letters in the superscript indicate significant differences in the same column (*p* <0.05).

Of particular interest is glutamate and glutamine metabolism, as glutamine is the most abundant amino acid in wheat proteins. Also, glutamine deamidation is essential for plant protein hydrolysis by glutamate, and glutamate-α-ketoglutarate interconversion by glutamate dehydrogenase provides amino receptors for the amino transfer of other amino acids (24). As shown (Table 2), glutamate was the most abundant amino acid (24–26%) while no glutamine was detected. When combined with secondary structure analyses, it appeared that only a very low level (2.66%) of the glutamine side chain was present in samples without WBDF, and that glutamine side chain content disappeared after WBDF addition. Therefore, WBDF addition may have caused a change in glutamine behavior. The exact mechanism requires further investigation.

### Surface hydrophobicity analysis

Surface hydrophobicity is a structural feature used to assess protein conformational changes. The exogenous fluorescence molecule ANS measures the surface hydrophobicity index (H_o_) of proteins; its values are positively correlated with hydrophobic group numbers on the surface of gluten and it provides an indication of protein-protein interactions. Most fluorescent aromatic amino acids are located in the protein interior, so if external forces unfold wheat gluten structures and expose hydrophobic groups (e.g., glutamine and asparagine) (25), this increases H_o_ values. Previously, the surface hydrophobicity of proteins was shown to increase or decrease after sourdough fermentation, and depended on the hydrolysis conditions of the system, the degree of hydrolysis, enzyme specificity, and protein properties (26).

**Figure 3:**
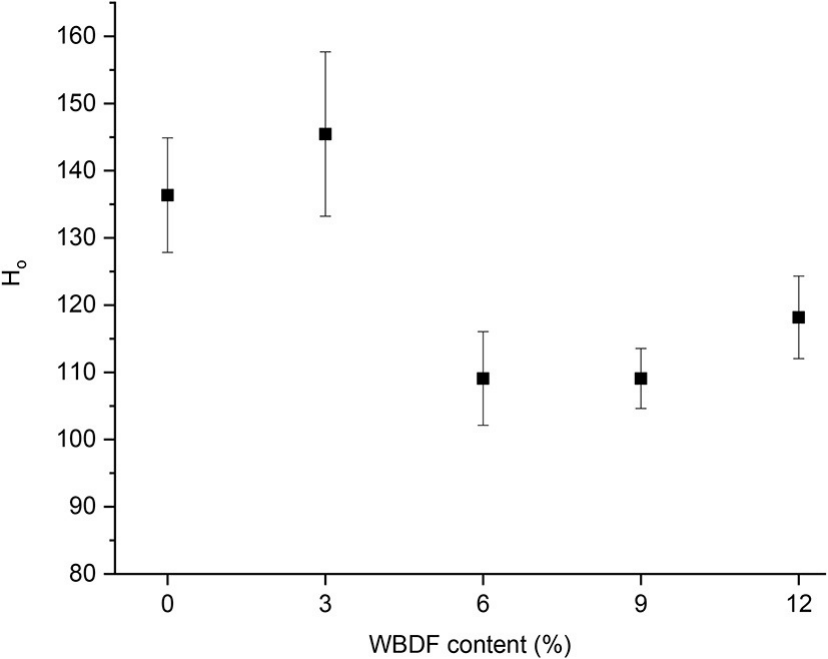
Surface hydrophobicity values of samples at different WBDF levels.

Changes in the surface hydrophobicity of gluten plus different WBDF concentrations after fermentation are shown (Figure 3); while H_o_ values in 3%-WBDF samples showed a slight increase when compared with control samples, this was not significant (*p* > 0.05). Additionally, a significant decrease in H_o_ was observed in 6, 9, and 12% WBDF samples (*p* < 0.05); this indicated that more hydrophilic amino acid groups appeared on the protein surface in the system, and a reduction in hydrophobicity on the gluten surface at high WBDF concentrations suggested possible depolymerization of gluten aggregates. However, no significant correlations between H_o_ changes and WBDF concentrations were identified (*p* > 0.05). Therefore, the effects of pH on peptide chains (fermentation processes) in the system cannot be excluded; acidic environments can affect hydrogen bond formation, electrostatic interactions, and hydrophobic interactions between protein molecules, leading to depolymerization, stretching of peptide chains, and hydrophobic group exposure. It should be noted that for alterations in protein secondary structures and free amino acid content, no strong evidence suggested that WBDF concentrations or the fermentation process dominated H_o_ changes.

### Thermal properties

**Figure 4:**
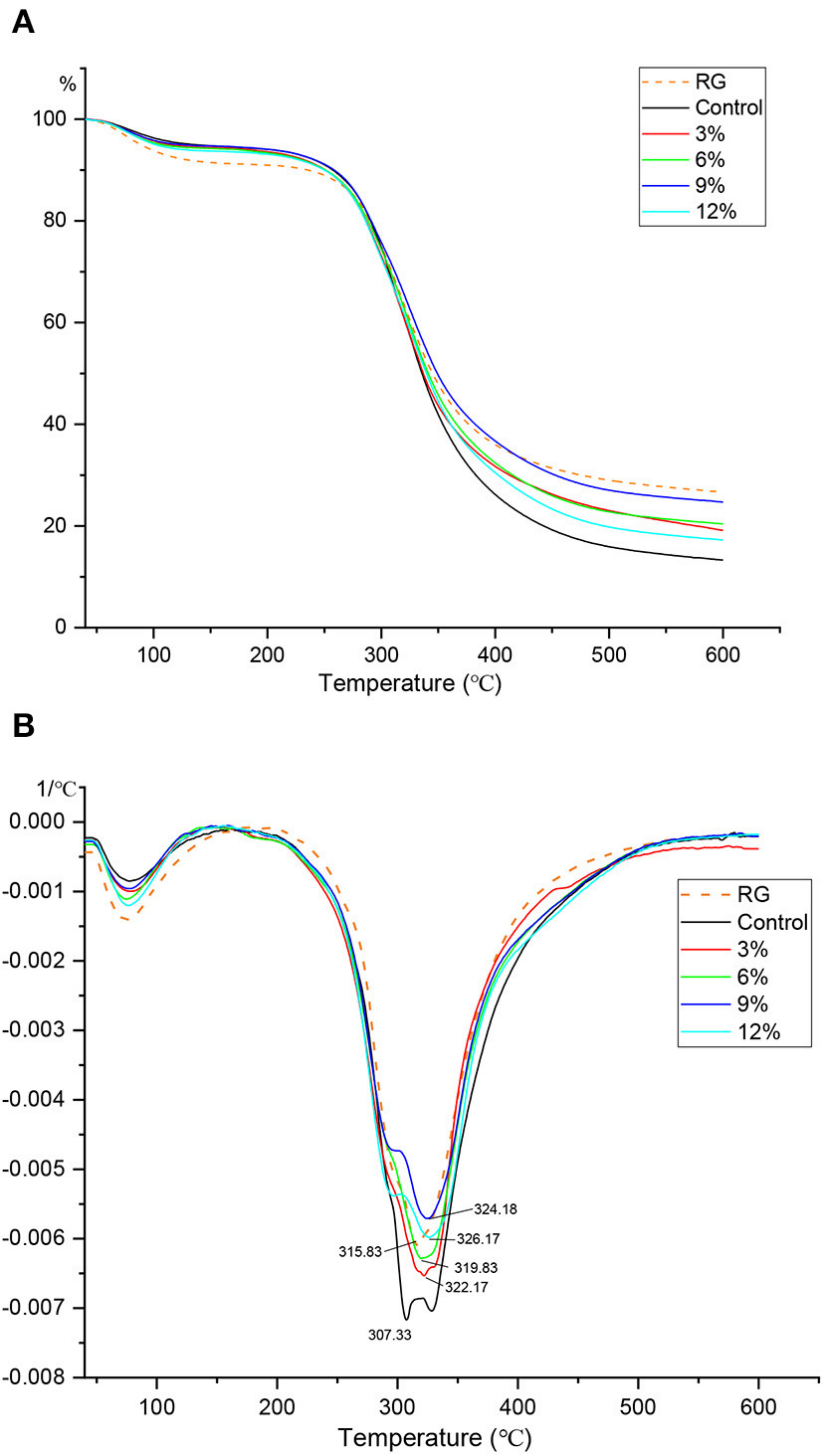
TGA **(A)** and DTG **(B)** profiles of different WBDF levels samples. RG is raw gluten unfermented.

TGA is commonly used to indicate how water evaporates from a system, and is used to investigate mechanisms whereby samples lose weight due to controlled heating. Weight loss curves also reflect variability between the behaviors of mixture components. Both TGA (Figure 4A) and DTG (Figure 4B, first derivative) curves for fermented gluten at different WBDF concentrations are indicated. As shown (Figure 4B), the turning point for sample mass loss occurred near 120°C, when it was hypothesized all free water in samples had evaporated. In a simple gluten mixture system, different changes in moisture content during warming can be distinguished by peak value changes in the DTG plot, which can be used to support TGA plot analysis. Specifically, the weight change at the first peak in the DTG curve (near 75°C) was possibly attributed to a reduction in free water content, with the second peak (307–324°C) occurring due to bound-water loss. During the first mass reduction interval, all samples containing WBDF showed no significant change when compared with the control; however, the fermentation process caused a slight increase in free water levels in samples when compared with RG samples. The thermal decomposition of the WBDF-gluten system was also divided into three stages. The mass loss phase in the first stage (T < 120°C) was mainly attributed to free water evaporation from the system; the mass loss in the second stage (120°C ≤ T ≤ 320°C) was attributed to starch decomposition; and the mass loss in the third stage (T > 320°C) was attributed to the full carbonization of samples. At the end of heating, curve-end analysis showed that the control sample had the lowest mass fraction at this point, while the opposite was true for RG sample, thereby confirming that fermentation increased the free water content in samples, with the increase most likely coming from semi-bound/bound water in the original sample. Additionally, the slightly higher mass fraction in WBDF samples when compared with controls may have been due to incomplete WBDF decomposition in the system, or extensive hydrogen bonding structures formed between WBDF and gluten/free amino acids, which increased thermal stability in samples.

## Conclusions

Investigating alterations in gluten after sourdough fermentation at different WBDF concentrations is required for an increased understanding and the production of whole grain and/or high fiber fermented flour products. We showed that WBDF addition fragmented gluten clusters during fermentation and disrupted gluten network continuity. Also, WBDF addition caused gluten polymers to be more readily depolymerized and reduced protein surface hydrophobicity; we hypothesized that non-covalent interactions of WBDF with glutamine side chains in gluten were the main cause of these observations. The ability of WBDF to influence the formation of flavor substance precursors; however, it should be taken into account that WBDF may affect the metabolic processes of glutamate and glutamine in the sourdough fermentation system. Finally, we hypothesize that the thermal properties of WBDF and its ability to hold water at physical layers were the main factors causing thermal property changes in the gluten system. This study provides insights on interactions between dietary fiber and gluten during fermentation, and provides an evidence-based guide for the production of fermented high fiber flour products.

## Data availability statement

The original contributions presented in the study are included in the article/Supplementary material, further inquiries can be directed to the corresponding author/s.

## Author contributions

ZW: conceptualization, methodology, software, and writing—original draft preparation. SM: conceptualization, writing—reviewing and editing, supervision, and project administration. LL: methodology, software, and visualization. JH: funding acquisition, supervision, and project administration. All authors contributed to the article and approved the submitted version.

## Funding

This work was supported by the National Natural Science Foundation of China (No. 32272249), Scientific and Technological Innovation Talents Project of Henan Universities (No. 23HASTIT033), Zhongyuan Scholars in Henan Province (No. 192101510004), Major Science and Technology Projects for Public Welfare of Henan Province (No. 201300110300), and open competition Research Projects of Xuchang University (No. 20220504).

## Conflict of interest

The authors declare that the research was conducted in the absence of any commercial or financial relationships that could be construed as a potential conflict of interest.

## Publisher's note

All claims expressed in this article are solely those of the authors and do not necessarily represent those of their affiliated organizations, or those of the publisher, the editors and the reviewers. Any product that may be evaluated in this article, or claim that may be made by its manufacturer, is not guaranteed or endorsed by the publisher.

## References

[1] Hu Y Ding M Sampson L Willett WC Manson JE Wang M. Intake of whole grain foods and risk of type 2 diabetes: results from three prospective cohort studies. BMJ. (2020) 370:m2206. 10.1136/bmj.m2206 32641435 PMC7341349

[2] Liu J Yu LL Wu Y. Bioactive components and health beneficial properties of whole wheat foods. J Agric Food Chem. (2020) 68:12904–15. 10.1021/acs.jafc.0c00705 32324395

[3] Reynolds AN Akerman AP Mann J. Dietary fiber and whole grains in diabetes management: systematic review and meta-analyzes. PLoS Med. (2020) 17:e1003053. 10.1371/journal.pmed.1003053 32142510 PMC7059907

[4] Ma S Wang Z Liu H Li L Zheng X Tian X. Supplementation of wheat flour products with wheat bran dietary fiber: purpose, mechanisms, and challenges. Trends Food Sci Technol. (2022) 123:281–9. 10.1016/j.tifs.2022.03.012

[5] Gobbetti M Gänzle M. Handbook on Sourdough Biotechnology. Springer Science and Business Media (2012).

[6] Ma S Wang Z Guo X Wang F Huang J Sun B. Sourdough improves the quality of whole-wheat flour products: mechanisms and challenges—A review. Food Chem. (2021) 360:130038. 10.1016/j.foodchem.2021.130038 34020364

[7] Suo B Chen X Wang Y. Recent research advances of lactic acid bacteria in sourdough: origin, diversity, and function. Curr Opin Food Sci. (2021) 37:66–75. 10.1016/j.cofs.2020.09.007

[8] Corsetti A Settanni L. Lactobacilli in sourdough fermentation. Food Res Int. (2007) 40:539–58. 10.1016/j.foodres.2006.11.001

[9] De Vuyst L Van Kerrebroeck S Leroy F. Microbial ecology and process technology of sourdough fermentation. Adv Appl Microbiol. (2017) 100:49–160. 10.1016/bs.aambs.2017.02.003 28732554

[10] Thiele C Grassl S Gänzle M. Gluten hydrolysis and depolymerization during sourdough fermentation. J Agric Food Chem. (2004) 52:1307–14. 10.1021/jf034470z 14995138

[11] Thiele C Gänzle MG Vogel RF. Contribution of sourdough lactobacilli, yeast, and cereal enzymes to the generation of amino acids in dough relevant for bread flavor. Cereal Chem. (2002) 79:45–51. 10.1094/CCHEM.2002.79.1.45

[12] Gobbetti M De Angelis M Di Cagno R Calasso M Archetti G Rizzello CG. Novel insights on the functional/nutritional features of the sourdough fermentation. Int J Food Microbiol. (2019) 302:103–13. 10.1016/j.ijfoodmicro.2018.05.018 29801967

[13] Xu D Ding W Ke W Li F Zhang P Guo X. Modulation of metabolome and bacterial community in whole crop corn silage by inoculating homofermentative *Lactobacillus plantarum* and heterofermentative *Lactobacillus buchneri*. Front Microbiol. (2019) 9:3299. 10.3389/fmicb.2018.03299 30728817 PMC6352740

[14] Heitmann M Zannini E Arendt E. Impact of Saccharomyces cerevisiae metabolites produced during fermentation on bread quality parameters: a review. Crit Rev Food Sci Nutr. (2018) 58:1152–64. 10.1080/10408398.2016.1244153 27874287

[15] Ma S Han W Li L Zheng X Wang X. The thermal stability, structural changeability, and aggregability of glutenin and gliadin proteins induced by wheat bran dietary fiber. Food Funct. (2019) 10:172–9. 10.1039/C8FO01810C 30516204

[16] Wang Z Yan J Ma S Tian X Sun B Huang J. Effect of wheat bran dietary fiber on structural properties of wheat starch after synergistic fermentation of *Lactobacillus plantarum* and *Saccharomyces cerevisiae*. Int J Biol Macromol. (2021) 190:86–92. 10.1016/j.ijbiomac.2021.08.179 34474052

[17] Wieser H Vermeulen N Gaertner F Vogel RF. Effects of different Lactobacillus and Enterococcus strains and chemical acidification regarding degradation of gluten proteins during sourdough fermentation. Eur Food Res Technol. (2008) 226:1495–502. 10.1007/s00217-007-0681-1

[18] Fraberger V Ladurner M Nemec A Grunwald-Gruber C Call LM Hochegger R. Insights into the potential of sourdough-related lactic acid bacteria to degrade proteins in wheat. Microorganisms. (2020) 8:1689. 10.3390/microorganisms8111689 33143014 PMC7693696

[19] Liu J Luo D Li X Xu B Zhang X Liu J. Effects of inulin on the structure and emulsifying properties of protein components in dough. Food Chem. (2016) 210:235–41. 10.1016/j.foodchem.2016.04.001 27211643

[20] Xu J Hao M Sun Q Tang L. Comparative studies of interaction of β-lactoglobulin with three polyphenols. Int J Biol Macromol. (2019) 136:804–12. 10.1016/j.ijbiomac.2019.06.053 31228500

[21] Zhang Y Hong T Yu W Yang N Jin Z Xu X. Structural, thermal and rheological properties of gluten dough: comparative changes by dextran, weak acidification and their combination. Food Chem. (2020) 330:127154. 10.1016/j.foodchem.2020.127154 32531630

[22] Gänzle MG Loponen J Gobbetti M. Proteolysis in sourdough fermentations: mechanisms and potential for improved bread quality. Trends Food Sci Technol. (2008) 19:513–21. 10.1016/j.tifs.2008.04.002

[23] Di Cagno R De Angelis M Auricchio S Greco L Clarke C De Vincenzi M. Sourdough bread made from wheat and nontoxic flours and started with selected lactobacilli is tolerated in celiac sprue patients. Appl Environ Microbiol. (2004) 70:1088–96. 10.1128/AEM.70.2.1088-1096.2004 14766592 PMC348803

[24] Tanous C Kieronczyk A Helinck S Chambellon E Yvon M. Glutamate dehydrogenase activity: a major criterion for the selection of flavor-producing lactic acid bacteria strains. Lactic Acid Bact Genet Metab Appl. (2002) 271–8. 10.1007/978-94-017-2029-8_17 12369193

[25] Pallarès I Vendrell J Avilés FX Ventura S. Amyloid fibril formation by a partially structured intermediate state of α-chymotrypsin. J Mol Biol. (2004) 342:321–31. 10.1016/j.jmb.2004.06.089 15313627

[26] De la Barca AC Ruiz-Salazar RA Jara-Marini ME. Enzymatic hydrolysis and synthesis of soy protein to improve its amino acid composition and functional properties. J Food Sci. (2000) 65:246–53. 10.1111/j.1365-2621.2000.tb15988.x

