# Supplementary material for: Effect of wheat bran dietary fiber on structural properties and hydrolysis behavior of gluten after synergistic fermentation of Lactobacillus plantarum and Saccharomyces cerevisiae
Source: Front Nutr. 2022 Sep 20;9:982878. doi: 10.3389/fnut.2022.982878 (PMC9530331; doi:10.3389/fnut.2022.982878)
Supplement: Supplementary file 1 [file Table_1.DOCX]

Table S1 Amide Ⅰ leads to the corresponding protein secondary structure belonging of each band range after convolution

| Band range (cm^-1^) | Protein secondary structure |
| --- | --- |
| 1594-1600 | Glutamine side chain |
| 1610-1614 | Intermolecular β-sheets |
| 1615-1637 | Antiparallel β-sheets |
| 1640-1645 | Random coils |
| 1650-1660 | α-helices |
| 1665-1681 | β-turns |
| 1682-1696 | β-sheets |

(a)


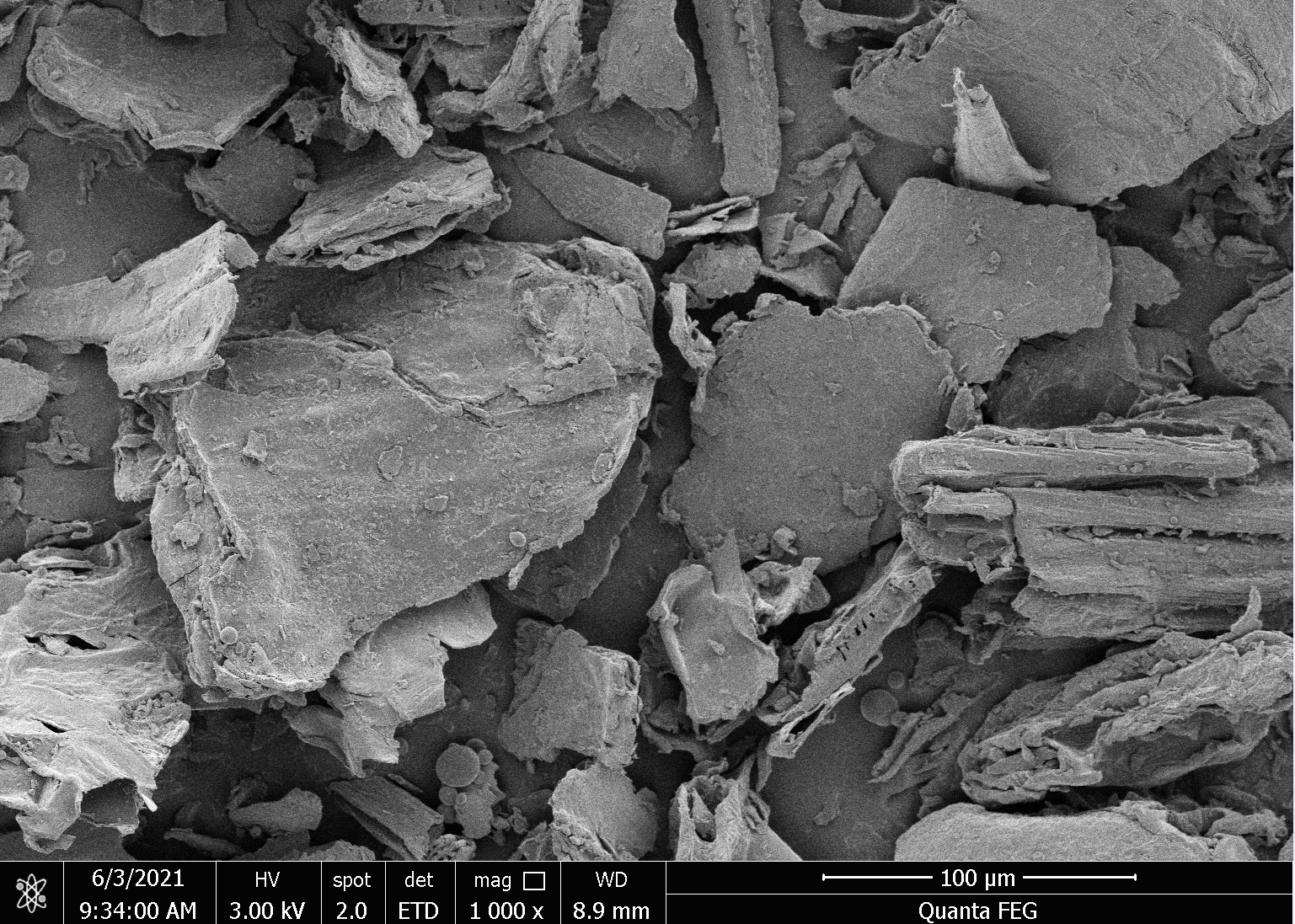


(b)


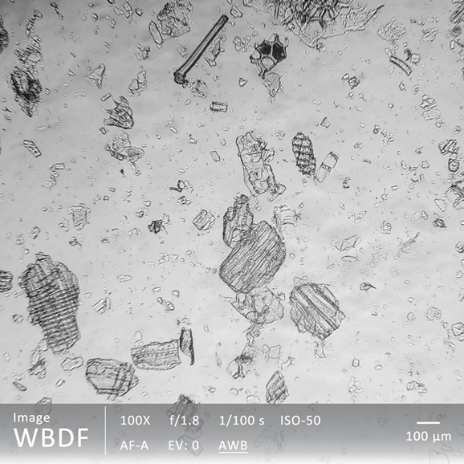


Figure S1 SEM image of WBDF (a) and optical microscopy image of WBDF (b)
